# Idiopathic Omental Bleeding Treated by Laparoscopic Partial Omentectomy: A Case Report and Review of the Literature

**DOI:** 10.7759/cureus.15795

**Published:** 2021-06-21

**Authors:** Shoryu Takayama, Koshiro Harata, Rei Mizuno, Riki Ganeko

**Affiliations:** 1 Surgery, Nagoya Tokushukai General Hospital, Nagoya, JPN; 2 Gastrointestinal Surgery, Nagoya City University East Medical Center, Nagoya, JPN; 3 Gastrointestinal Surgery, Uji Tokushukai Hospital, Kyoto, JPN

**Keywords:** idiopathic omental bleeding, laparoscopic surgery, intra-abdominal bleeding, omentectomy, ivr

## Abstract

Omental bleeding is potentially life-threatening. There are many causes of omental bleeding including trauma, neoplasia, arterial aneurysm rupture, omental torsion, vasculitis, or segmental arterial mediolysis (SAM). Without remarkable pathological features, the diagnosis of idiopathic omental bleeding is made. Omental bleeding is relatively a rare disease, and there is no established treatment strategy.

A 53-year-old woman was brought to the ED for sudden onset abdominal pain. CT revealed hematoma in the omentum and was diagnosed as idiopathic omental bleeding accordingly. The patient underwent laparoscopic partial omentectomy and was discharged nine days after surgery. The pathological findings of the resected omentum were not remarkable, and the final diagnosis was made as idiopathic omental bleeding.

In some case reports of omental bleeding, interventional radiology (IVR) was chosen for hemostasis, but IVR cannot resect tissue of omentum so it is difficult to make a pathological diagnosis. The surgical approach of idiopathic omental bleeding is uncommon. However, the use of the laparoscopic approach hasn't been reported in the literature. Laparoscopic partial omentectomy can provide effective hemostasis. We report laparoscopic partial omentectomy surgical procedure and review of the literature.

## Introduction

Omental bleeding results from trauma, neoplasia [1], arterial aneurysm rupture [2], omental torsion [3], vasculitis [4], or segmental arterial mediolysis (SAM) [5]. Without remarkable pathological features, the diagnosis of idiopathic omental bleeding is made [6]. Omental bleeding is potentially life-threatening, so it is necessary to provide effective hemostasis. Omental bleeding is a relatively rare disease, and there is no established treatment strategy. In recent years, interventional radiology (IVR) provided effective hemostasis in some cases of omental bleeding [7], but IVR cannot resect tissue of omentum so it is difficult to make a pathological evaluation. In some case reports, surgical treatment was chosen, but laparoscopic surgical treatment is not reported.

## Case presentation

**Figure 1:**
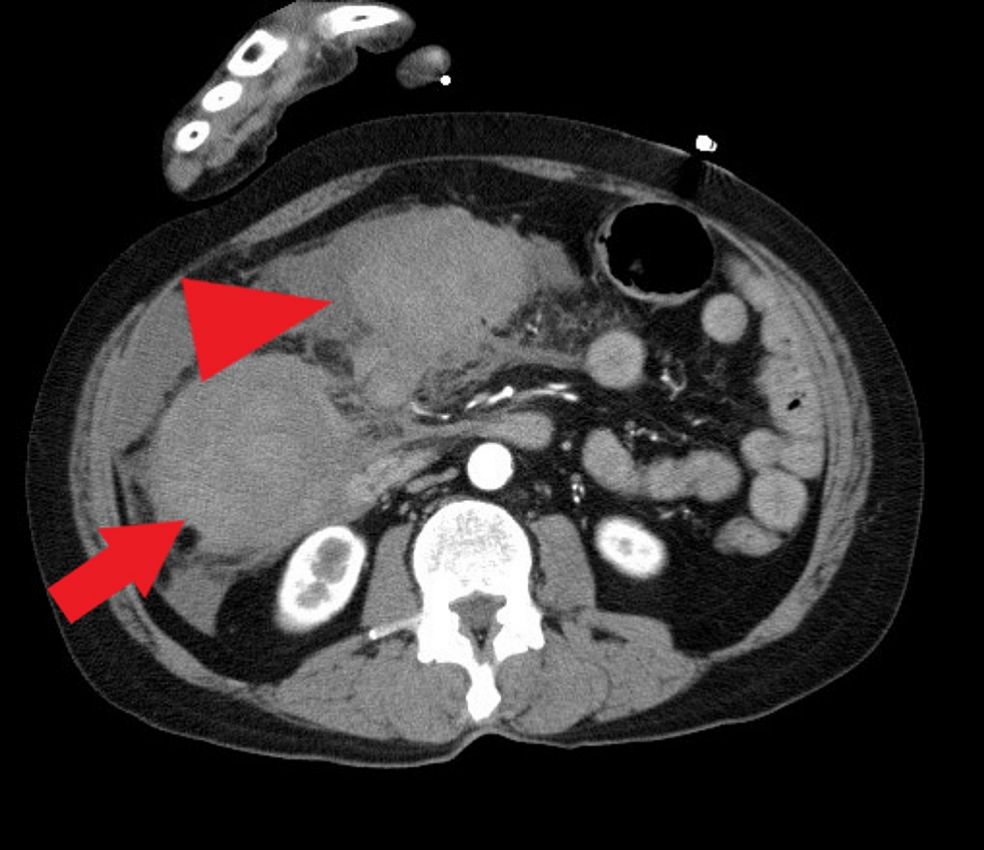
Preoperative contrast CT. Contrast CT showed hemorrhage ascites in the extrahepatic region (arrow) and hematoma in the omentum and omental bursa (arrowhead).

A 53-year-old woman, who is a known case of rheumatoid arthritis, was brought to the ED complaining of sudden onset right upper quadrant pain one hour ago without any trauma history. Her medications include methotrexate and methylprednisolone. Her surgical history included appendicectomy 35 years ago and cesarean section 20 years ago. On physical examination, her blood pressure was 135/86 mmHg and pulse was 80/min. There was tenderness in the right upper quadrant, and her abdomen was rigid. Laboratory studies showed a hemoglobin level of 11.8 g/dl, white blood cell count of 7700 /μl, platelet count of 24.2 × 10^4 ^μl, and other data were within normal range. Contrast-enhanced CT showed hemorrhagic ascites in the extrahepatic region and hematoma in the omentum and omental bursa with no extravasation (Figure 1). As a result, a preoperative diagnosis of omental bleeding was established. There was no evidence of active bleeding and her vital signs were stable, therefore we performed laparoscopic partial omentectomy.

**Figure 2:**
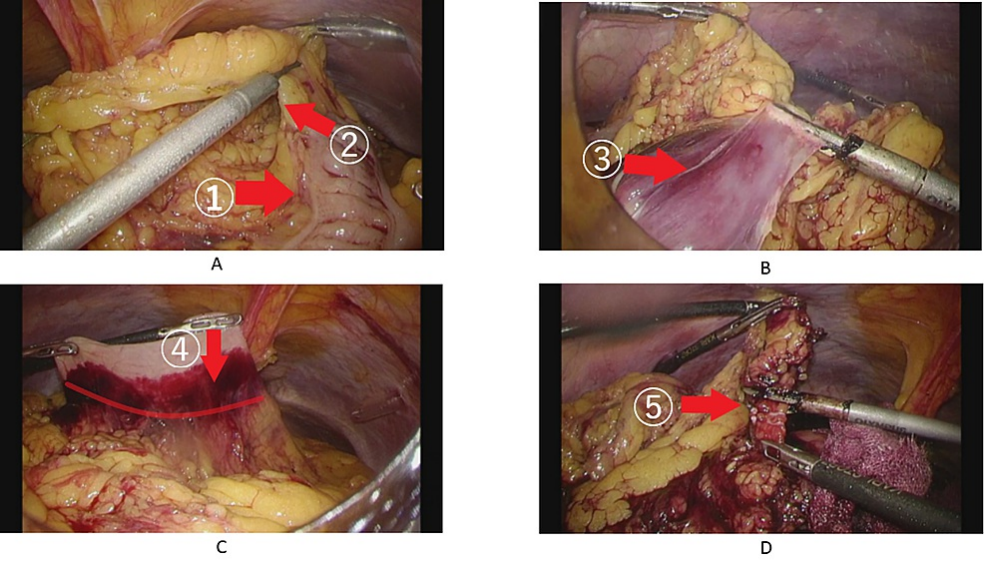
Surgical procedure. [A] The omentum was separated from the attachment to the transverse colon ① and the omental bursa ② was opened. [B] The omentum was separated to the right border of the omental bursa taking care not to damage the mesentery of the transverse colon ③. [C] The omentum was separated from the gastric wall ④. [D] The right gastroepiploic artery and vein (RGEA.V) ⑤ were clipped and cut.

First, the omentum was separated from the attachment to the transverse colon and the omental bursa was opened (Figure 2A). The omentum was cut to the right border of the omental bursa, taking care not to damage the mesentery of the transverse colon (Figure 2B). Next, the omentum was separated from the gastric wall (Figure 2C). Then the right gastroepiploic artery and vein (RGEA.V) were clipped and dissected (Figure 2D). Omentum tissue was then dissected and the specimen was removed. Finally, 6.5-mm continuous suction drains were placed by the extrahepatic cavity, left subdiaphragmatic cavity, and Douglas fossa. Because there were no remarkable pathological findings in the removed omentum, the diagnosis of idiopathic omental bleeding was made. The postoperative course was very good, and the patient was discharged nine days after surgery. After three months, she came to the outpatient clinic and her laboratory studies were within normal range and ultrasonography showed no ascites and hematoma.

## Discussion

Omental bleeding is a relatively rare disease, and there is no established treatment strategy. We searched for case reports about omental bleeding and found 25 cases (Table 1). Treatments were reported as transcatheter arterial embolization (TAE) or laparotomy. TAE can provide effective hemostasis when rapid hemostasis is required. However, there was a case in which partial omentectomy was performed for pathological evaluation after TAE [8]. Pathological evaluation is necessary because some omental bleedings result from neoplasia, arterial aneurysm rupture, omental torsion, vasculitis, and SAM. If omental bleeding is diagnosed preoperatively and patient status is stable, surgery may be a good choice because it can provide effective hemostasis and resect tissue of omentum simultaneously. All cases of omental bleeding treated by surgery were performed by laparotomy. These reported cases underwent either ligation or partial omentectomy. In this case report, we performed laparoscopic partial omentectomy. Although there is no report of laparoscopic partial omentectomy, this procedure can be performed for patients with stable preoperative vital signs and no extravasation like our case. For example, patients whose postoperative diagnosis was idiopathic omental bleeding or omental torsion (Table 1) might be suitable for a laparoscopic partial omentectomy approach, because their vital signs were stable.

**Table 1:** Omental bleeding case reports. NA: Not available; NR: Not remarkable; IVR: Interventional radiology; SAM: Segmental arterial mediolysis; TAE: Transcatheter arterial embolization.

Case	First author	Year	Age/Gender	Chief complaint	Shock vital	Examination		Therapy/Surgical technique	Pathology
						CT	IVR		
1	Leitner MJ et al. [3]	1950	49/M	Abdominal pain		NA	-	Laparotomy	NR
2		1951	52/F	Pain in the right lower quadrant of the abdomen		NA	-	Laparotomy	NR
3		1949	51/F	Right upper abdominal pain, nausea, and vomiting		NA	-	Laparotomy	NR
4		1951	27/M	Discomfort in the right upper quadrant of the abdomen		NA	-	Laparotomy	NR
5		1945	82/M	Abdominal pain, vomiting, and nausea		NA	-	Laparotomy	NR
6		1950	64/M	Abdominal pain		NA	-	Laparotomy	NR
7	Heritz DM et al. [5]	1990	68/M	Abdominal pain	Shock vital	〇	〇	Laparotomy partial omentectomy	SAM
8	Kroot EJ et al. [4]	2003	70/M	Abdominal pain		〇	-	Laparotomy partial omentectomy	Wegener granulomatosis
9	Jadav M et al. [2]	2004	60/M	Acute abdomen, nausea, vomiting, and diarrhea		〇	-	Laparotomy	NA
10	Finly DS et al. [9]	2005	41/M	Abdominal pain		〇	-	Laparotomy ligation of the omental varix	NA
11	Ohno T et al. [10]	2005	27/M	Intermittent abdominal pain		〇	-	Laparotomy partial omentectomy	NR
12	Nagaba Y et al. [11]	2005	64/M	Acute abdomen	Shock vital	〇	〇	TAE	-
13	Matsumoto T et al. [8]	2010	25/M	Abdominal pain		〇	〇	TAE → Laparotomy partial omentectomy	NR
14	Henry D and Satgunam S [6]	2012	24/F	Malaise, myalgias, and fatigue		〇	-	Laparotomy-only ligation	-
15	Takahashi M et al. [7]	2012	27/M	Abdominal pain, feeling faint		〇	〇	TAE	-
16	Cheng VE et al. [12]	2014	68/M	Acute hypotension, severe left abdominal pain	Shock vital	〇	-	Laparotomy	NA
17	Aumann V et al. [13]	2016	20/M	NA		〇	-	Laparotomy	NR
18	Kimura J et al. [14]	2016	29/M	Abdominal pain		〇	-	Laparotomy partial omentectomy	NR
19	Lyu YX et al. [15]	2018	58/M	Left upper quadrant pain		〇	-	Laparotomy partial omentectomy	NR
20	Mahmoudi A et al. [1]	2020	3/M	Abdominal distention		〇	-	Laparotomy partial omentectomy	Lymphangioma
21	Nishiyama T et al. [16]	2020	55/M	Acute abdomen		〇	〇	TAE	-
22			60/M	Acute abdomen		〇	〇	TAE	-
23	Moriarty HK et al. [17]	2020	60/M	Feeling faint	Shock vital	〇	〇	TAE	-
24			37/F	Abdominal pain, feeling faint	Shock vital	〇	〇	TAE	-
25			69/F	Abdominal pain		〇	〇	TAE	-

## Conclusions

Idiopathic omental bleeding is a relatively rare disease, and laparoscopic surgical approach was not reported in the literature. Laparoscopic partial omentectomy can provide effective hemostasis and pathological evaluation. When a patient is stable and without extravasation, laparoscopic partial omentectomy may be the first choice to treat.
